# Development of a Melanoma Risk Prediction Model Incorporating MC1R Genotype and Indoor Tanning Exposure: Impact of Mole Phenotype on Model Performance

**DOI:** 10.1371/journal.pone.0101507

**Published:** 2014-07-08

**Authors:** Lauren A. Penn, Meng Qian, Enhan Zhang, Elise Ng, Yongzhao Shao, Marianne Berwick, DeAnn Lazovich, David Polsky

**Affiliations:** 1 The Ronald O. Perelman Department of Dermatology, New York University School of Medicine, New York City, New York, United States of America; 2 Department of Population Health and Environmental Medicine, New York University School of Medicine, New York City, New York, United States of America; 3 Division of Epidemiology and Biostatistics, Department of Internal Medicine, University of New Mexico Cancer Center, Albuquerque, New Mexico, United States of America; 4 Division of Epidemiology and Community Health, School of Public Health, Masonic Cancer Center, University of Minnesota, Minneapolis, Minnesota, United States of America; IPATIMUP/Faculty of Medicine of the University of Porto, Portugal

## Abstract

**Background:**

Identifying individuals at increased risk for melanoma could potentially improve public health through targeted surveillance and early detection. Studies have separately demonstrated significant associations between melanoma risk, melanocortin receptor (MC1R) polymorphisms, and indoor ultraviolet light (UV) exposure. Existing melanoma risk prediction models do not include these factors; therefore, we investigated their potential to improve the performance of a risk model.

**Methods:**

Using 875 melanoma cases and 765 controls from the population-based Minnesota Skin Health Study we compared the predictive ability of a clinical melanoma risk model (Model A) to an enhanced model (Model F) using receiver operating characteristic (ROC) curves. Model A used self-reported conventional risk factors including mole phenotype categorized as “none”, “few”, “some” or “many” moles. Model F added MC1R genotype and measures of indoor and outdoor UV exposure to Model A. We also assessed the predictive ability of these models in subgroups stratified by mole phenotype (e.g. nevus-resistant (“none” and “few” moles) and nevus-prone (“some” and “many” moles)).

**Results:**

Model A (the reference model) yielded an area under the ROC curve (AUC) of 0.72 (95% CI = 0.69, 0.74). Model F was improved with an AUC = 0.74 (95% CI = 0.71–0.76, p<0.01). We also observed substantial variations in the AUCs of Models A & F when examined in the nevus-prone and nevus-resistant subgroups.

**Conclusions:**

These results demonstrate that adding genotypic information and environmental exposure data can increase the predictive ability of a clinical melanoma risk model, especially among nevus-prone individuals.

## Introduction

With a dismal overall survival for advanced disease, cutaneous melanoma results in more years-of-life-lost than any major adult cancer besides breast [1]. Fortunately, melanoma is highly curable when diagnosed at its earliest stages [2], and a recent population-based skin cancer screening program in Germany successfully demonstrated a decrease in melanoma mortality [3]; however, due to the relative rarity of melanoma compared to other cancers, like breast and prostate, implementing a population-based screening initiative can be expensive and impractical. Thus, refining such programs to prioritize patients at highest risk for the disease may improve the cost-effectiveness of such activities [4].

Although well-established melanoma risk factors exist (e.g. fair pigmentation, increased numbers of nevi, excessive outdoor ultraviolet (UV) light exposure), analysis of case-control studies demonstrate that as many as 50% of melanoma patients lack these risk factors, hindering our ability to identify those at greatest risk [5]–[7]. Additionally, certain relevant risk factors may be more common in different patients who develop different melanoma subtypes (i.e. superficial spreading, nodular, lentigo maligna, etc.). For example, patients who develop superficial spreading melanoma are more likely to have an increased number of nevi, while lentigo maligna melanoma is more common in individuals with chronic sun damage. These clinical patterns have been synthesized into a unifying concept suggesting that melanoma may result from different causal pathways (i.e. The Divergent Pathway Theory). Specifically, these studies describe two causal pathways of melanomagenesis associated with different UV exposure patterns and patient nevus phenotypes [8], the latter of which are known to have a strong genetic component [9], [10]. This may explain why existing melanoma risk assessment tools have failed to reach the mainstream clinical arena, and suggests the need to account for this disease heterogeneity to maximize their accuracy.

Fortunately, new risk factors have been described, including genetic markers and indoor tanning exposure, which may improve early detection and screening efforts [11]–[15]. Indoor UV exposure, which is associated with a 59% increase in melanoma risk when exposure occurs before the age of 35 [16], [17], is of particular concern as approximately 27% of women under the age of 35 engage in indoor tanning in the United States [18]. Among genetic factors associated with melanoma, polymorphisms in the melanocortin-1 receptor (MC1R) are common and exhibit moderate penetrance. MC1R is a key regulator of melanin synthesis, playing a major role in hair and skin pigmentation [19]. MC1R is also involved in UV-induced DNA repair [20]; and several of its 80 known variants are associated with melanoma risk [19], [21].

What has yet to be determined is whether genetic markers and indoor UV exposure can improve the performance of a clinically-based melanoma risk prediction model. Our primary objective of this study, therefore, was to determine if adding a genetic marker (e.g. MC1R genotype) and indoor UV exposure measures to a clinically-based melanoma risk assessment model could improve its predictive ability. A secondary objective was to investigate the model's performance when stratified by patient subpopulations defined by nevus phenotype, as a way to assess the possible effect of melanoma heterogeneity in risk prediction models.

## Materials and Methods

### Study Subjects

The current study, based on information collected from the Minnesota Skin Health Study (SHS), and was deemed exempt from review by the IRB at New York University Langone Medical Center because a) our study consisted of existing data, and b) these data did not include personal identifying information.

The data analyzed in this study are based on information collected from a subset of subjects from the SHS, a study approved by the Institutional Review Board (IRB) at the University of Minnesota. This population-based case control study evaluated the association between outdoor and indoor UV exposure and melanoma risk [11]. Briefly, cases were accrued through the Minnesota Cancer Surveillance System, the state's cancer registry. Individuals' aged 25 to 59 diagnosed with invasive cutaneous melanoma, of any histologic subtype, between July 2004 and December 2007 were eligible for enrollment. Controls were randomly selected from the Minnesota state driver's license list and matched to cases in a 1∶1 ratio on age (in 5-year age groups) and gender. Cases and controls were required to be English-speaking and to have a telephone number. In addition to providing mouthwash samples, subjects received a self-administered questionnaire, from which selected information was used to facilitate a detailed 1-hour telephone interview that collected information pertaining to indoor tanning, outdoor sun exposure measures, sunscreen use, family history of melanoma, and host characteristics (hair, eye, and skin color; freckling; and mole phenotype). Nevus/mole phenotype was assessed using cartoon diagrams illustrating 4 categories of nevus density (“none”, “few”, “some”, or “many” moles). For the purpose of this study, subjects who reported having “none” or “few” moles were categorized as “nevus-resistant”, while those with “some” or “many” moles were categorized as “nevus-prone” (Figure S1). Further description of materials and methods from the SHS, including assessment of bias and questionnaire development, can be found in the original publication [11].

### MC1R Genotyping

Based on availability of germ line DNA, extracted from mouthwash specimens, our analytic sample was comprised of 1640 subjects from the SHS. MC1R genotyping was performed via Sanger sequencing of the entire coding region of MC1R. Variants were detected using Mutation Surveyor Software and confirmed by visual inspection.

We based the categorization of MC1R variants on the results of a meta-analysis by Whiteman et al. [13]. Variants strongly associated with red hair color (RHC) included D84E, R151C, R160W, and D294H were designated as “R”. Variants weakly associated with red hair color (NRHC) included V60L, V92M, R142H, I155T, and R163Q were denoted “r”. [13], [14]. Historically, studies have not addressed the appropriate assignment of rare variants and insertions/deletions (indels) as “R” or “r” [13], [14], [22]. We assigned indels and rare variants as “R” or “r” based on their association with melanoma case-control status using a two degree-of-freedom chi-square test (Pearson or Fisher Exact) for genotypes. We also assessed their melanoma odds ratios when combined into genotypes with consensus, “R”, and “r” alleles. The specific assignments of these genotypes are described in the Results.

### Statistics/Model Building

To identify the host and environmental exposure characteristics collected by the SHS that were most appropriate for inclusion in a risk prediction model, we first applied univariate logistic regression model on each measure to obtain the unadjusted odds ratio and the corresponding p-value from the Wald test, in order to select the characteristics significantly associated with melanoma case-control status (p-value<0.05). As the categorical measures of UV exposure are all ordinal variables, we applied the same univariate logistic regression model regarding each ordinal measure as a continuous variable. We also calculated the melanoma odds ratios (OR) for each characteristic. Of the measures significantly associated with melanoma, those chosen for the development of our risk prediction model included a) host characteristics used in previously developed melanoma risk models, b) outdoor UV exposure measures, and c) indoor UV exposure measures.

We used multivariate logistic regression to calculate odds ratios (OR) and 95% confidence intervals (CI) to determine the degree of melanoma risk associated with 1) selected host and environmental risk factors, 2) and MC1R genotypes.

The first model (Model A) was based only on host risk factors. We created additional models (Models B–E) that included different combinations of UV exposure measures (outdoor and indoor) and/or MC1R genotype to assess their effect on the predictive ability of the clinical model. For Model F, we combined UV exposure measures and MC1R genotype categories together with the host risk factors in Model A.

We compared the discriminative power of the risk indices using the area under the Receiver Operating Characteristic (ROC) curve metric. DeLong's test was used to test the significance of the incremental increase of AUCs under the ROC curves between Models B through F and the baseline Model A. We evaluated the models' calibration using the Hosmer-Lemeshow test. The model was cross-validated to obtain the 95% confidence interval of the area under the ROC curve (AUC). Seventy five percent of data was randomly chosen as the training sample to develop the model. This model, with the same regression coefficients, was then applied to the remaining 25% of data to assess the AUC of the model. This procedure was repeated 10,000 times, resulting in a sample of 10,000 estimated AUC's. The 95% confidence interval (CI) of the AUC was based on the 2.5% percentile and 97.5% percentile of the set of estimated AUCs. We then analyzed model performance in subgroups stratified by mole phenotype to assess variation in the models' discriminative ability in subjects with and without an increased number of nevi.

## Results

### Patient Characteristics and their Association with Melanoma Risk

#### Phenotypic and Environmental Risk Factors Associated with Melanoma

Host information and MC1R sequencing data were available for 1640 subjects. Cases and controls had a similar mean age and gender distribution. Several host characteristics were significantly associated with melanoma risk such as light pigmentation, freckling, and an increased number of nevi (detailed results shown in Table 1). Our study group is representative of the complete sample of cases and controls from the SHS study, as demonstrated in our comparison of participant characteristics in Table 1.

**Table 1:** Participant Characteristics.*

Variables	Analytic Sample (N = 1640)		Unadjusted Odds Ratio (95% CI)	P-value	Adjusted Odds Ratio** (95% CI)	P-value	SHS Cohort (N = 2268)	
	Cases N (%)	Controls N (%)					Cases N (%)	Controls N (%)
Gender								
Female	526 (60.1)	442 (57.8)	Ref		Ref		699 (59.9)	656 (59.6)
Male	349 (39.9)	323 (42.2)	0.91 (0.75–1.11)	0.34	1.21 (0.95–1.53)	0.12	468 (40.1)	445 (40.3)
Age (Mean)	46.5	46.2		0.12	1.03 (1.02–1.04)	<0.01	46.2	45.8
Eye color								
Brown	176 (20.1)	187 (24.4)	Ref		Ref		226 (19.4)	278 (25.2)
Hazel	179 (20.5)	158 (20.7)	1.20 (0.90–1.62)	0.22	0.88 (0.65–1.20)	0.43	237 (20.3)	236 (21.4)
Green	136 (15.5)	102 (13.3)	1.42 (1.02–1.97)	0.04	0.97 (0.66–1.41)	0.86	175 (15.0)	142 (12.9)
Blue/Grey	384 (43.9)	318 (41.6)	1.28 (1.00–1.65)	0.05	1.00 (0.72–1.40)	0.98	529 (45.3)	445 (40.4)
Hair Color								
Dark brown/black	217 (24.8)	268 (35.0)	Ref		Ref		289 (24.8)	391 (35.5)
Light brown	305 (34.9)	308 (40.3)	1.22(0.96–1.55)	0.10	1.11 (0.84–1.45)	0.46	396 (33.9)	438 (39.8)
Blonde	260 (29.7)	163 (21.3)	1.97 (1.51–2.57)	<0.01	1.79 (1.30–2.46)	<0.01	362 (31.0)	226 (20.5)
Red	93 (10.6)	26 (3.4)	4.42 (2.76–7.07)	<0.01	4.38 (2.50–7.67)	<0.01	120 (10.3)	46 (4.2)
Skin Color								
Dark olive to black	6 (0.7)	25 (3.3)	Ref		Ref		11 (0.9)	36 (3.3)
Light olive	82 (9.4)	128 (16.7)	Ref (combined)		Ref (combined)		114 (9.8)	191 (17.3)
Fair	634 (72.5)	521 (68.1)	2.12 (1.59–2.82)	<0.01	1.57(1.12–2.18)	0.01	827 (70.9)	746 (67.8)
Very fair	153 (17.5)	91 (11.9)	2.92 (2.02–4.23)	<0.01	1.96 (1.26–3.06)	<0.01	215 (18.4)	128 (11.6)
Freckling								
None	404 (46.3)	453 (59.5)	Ref		Ref		547 (47.1)	635 (58.0)
Very few	257 (29.4)	195 (25.6)	1.48 (1.18–1.86)	<0.01	1.30 (1.01–1.68)	0.04	326 (28.1)	278 (25.4)
Few	145 (16.6)	82 (10.8)	1.98 (1.47–2.68)	<0.01	1.35 (0.96–1.90)	0.09	196 (16.9)	127 (11.6)
Some/Many	67 (7.7)	31 (4.1)	2.42 (1.55–3.79)	<0.01	1.43 (0.84–2.45)	0.19	93 (8.0)	55 (5.0)
Moles								
None	144 (16.5)	292 (38.2)	Ref		Ref		191 (16.5)	446 (40.7)
Few	492 (56.2)	393 (51.4)	2.54 (2.00–3.23)	<0.01	2.84 (2.18–3.69)	<0.01	644 (55.7)	545 (49.8)
Some/Many	239 (27.3)	80 (10.4)	6.06 (4.39–8.36)	<0.01	7.22 (5.05–10.30)	<0.01	321 (27.7)	104 (9.5)
Lifetime Sunburns								
None	17 (1.9)	42 (5.5)	Ref		Ref		33 (2.8)	68 (6.2)
1–2	119 (13.6)	147 (19.3)	2.00 (1.08–3.69)	0.03	1.26 (0.65–2.46)	0.49	168 (14.4)	221 (20.1)
3–5	174 (19.9)	143 (18.7)	3.01 (1.64–5.51)	<0.01	1.66 (0.85–3.21)	0.13	224 (19.2)	215 (19.6)
>5	564 (64.5)	431 (56.5)	3.23 (1.81–5.76)	<0.01	1.60 (0.85–3.01)	0.15	739 (63.5)	595 (54.1)
Indoor UV exposure								
Never	326 (37.6)	377 (50.4)	Ref		Ref		433 (39.8)	538 (53.0)
≤10 hours	256 (29.5)	206 (27.5)	1.44 (1.14–1.82)	<0.01	1.40 (1.07–1.83)	0.01	274 (25.2)	243 (23.9)
>10 hours	285 (32.9)	165 (22.1)	2.00 (1.57–2.55)	<0.001	2.32 (1.73–3.11)	<0.01	382 (35.1)	234 (23.1)

*For some of the variables, the total count does not equal 1640 due to a small number of missing values.

** Adjusted for gender, age, eye color, hair color, skin color, freckling, moles, lifetime sunburns, and hours of indoor tanning.

#### Environmental Risk Factors

We separately analyzed 52 measures of lifetime and decade-specific sun exposure [23], indoor tanning exposure and sunburn history, many of which were significantly associated with melanoma risk (Tables S1 & S2 in File S1) [11]. The incorporation of too many predictor variables could over fit and negatively impact the integrity of the model [24]. We therefore selected environmental characteristics with the most appropriate association with melanoma case-control status, which we based on statistical significance and melanoma odds ratios (OR). The two factors that met our inclusion criteria were “lifetime number of outdoor sunburns”, and “frequency (hours) of indoor tanning”. Subjects with no indoor UV exposure were assigned to the category of 0 hours of indoor tanning.

Indoor tanning frequency was measured several ways (Table S2 in File S1), nearly all of which were significant on univariate analysis. We considered hours of indoor tanning to be the most appropriate measure, as it minimizes interpersonal variation associated with the number and duration of individual indoor tanning sessions. There was a significant dose-response relationship between hours of indoor tanning and melanoma risk, with greater than 10 hours of indoor tanning associated with an adjusted OR of 2.32 (95% CI 1.73–3.11; p<0.01) in multivariate analysis (Table 1). In contrast, lifetime number of sunburns was only associated with a significant increase in melanoma risk in univariate analysis, but not multivariate analysis. Greater than 5 sunburns was associated with an OR of 3.23 (95% CI = 1.81–5.76; p<0.01) in univariate analysis and 1.60 (95% CI = 0.85–3.01; p = 0.15) multivariate analysis.

### Assignment of MC1R Genotype

Table 2 displays the variant frequencies of the 9 most common MC1R polymorphisms. The remaining non-synonymous polymorphisms were categorized as rare variants. Indels comprised their own category. We investigated the most appropriate categorization of indels and rare variants by combining them into genotypes with consensus, “R” or “r” variants (Table 3). Rare variants, when combined into genotypes with “R” or “r” alleles, were associated with a melanoma OR of 6.63 (95% CI = 1.89–23.27, p<0.01). Due to their strong association with melanoma case-control status, we classified rare variants as “R” alleles. Similarly, we also classified indels as “R” alleles, as genotypes that combined indels with consensus alleles had a melanoma OR of 3.65 (95% CI = 1.14–11.65, p = 0.03), and indels in genotypes with “R”, “r”, or “rare” had an OR of 7.29 (95% CI = 1.59–33.34, p = 0.01). In Table 4, we show the melanoma odds ratios of the assigned MC1R genotypes. Subjects with “r/r” or genotypes containing “R” alleles had statistically significantly higher risk of melanoma compared to subjects with “consensus” genotypes, with the “R/R” genotype carrying the greatest melanoma risk with an OR of 4.31 (95% CI; 2.69–6.89; p<0.01).

**Table 2:** MC1R Variant Allele Frequency.*

MC1R Variants	Cases N (%)	Controls N (%)	P-value**	Allele Dominance (R/r)
**V60L**	192 (21.94)	146 (19.08)	0.17	r
**V92M**	138 (15.77)	129 (16.86)	0.60	r
**R163Q**	98 (11.20)	94 (12.29)	0.54	r
**R142H**	11 (1.26)	10 (1.31)	0.90	r
**I155T**	18 (2.06)	6 (0.78)	0.05	r
**D84E**	30 (3.43)	18 (2.35)	0.25	R
**R151C**	205(23.43)	114 (14.90)	<0.01	R
**R160W**	173 (19.77)	121 (15.82)	0.04	R
**D294H**	46 (5.26)	19 (2.48)	0.01	R
**Indels**	22 (2.51)	6 (0.78)	0.01	R
**Rare**	26 (2.97)	14 (1.83)	0.18	R

*Allele frequency is determined from the total number of chromosomes genotyped.

** Chi^2^ or Fischer's exact test, as appropriate.

**Table 3:** Melanoma Risk of Genotypes Combining Indels and Rare Variants with Conventional MC1R Variants.

Genotype	Cases (n = 875)	Controls (n = 765)	Unadjusted OR (95% CI)	P-value
**Consensus/consensus**	163	216	Ref	
**rare/consensus**	10	11	1.21 (0.50–2.91)	0.68
**Indel/consensus**	11	4	3.65 (1.14–11.65)	0.03
**rare/(R or r)**	15	3	6.63 (1.89–23.27)	<0.01
**Indel/(r, R, or rare)**	11	2	7.29 (1.59–33.34)	0.01

“R”: D84E, R151C, R160W, D294H.

“r”: V60L, V92M, R163Q, R142H, I155T.

**Table 4:** Melanoma Risk Based on MC1R Genotyping including Indels and Rare Variants.

Genotype	Cases (n = 875)	Controls (n = 765)	Unadjusted OR (95% CI)	P-value
**Consensus/consensus**	163	216	Ref	
**r/consensus**	203	220	1.22 (0.93–1.62)	0.18
**r/r**	67	55	1.61 (1.07–2.43)	0.03
**R/Consensus**	208	172	1.60 (1.20–2.13)	<0.01
**R/r**	143	74	2.56 (1.81–3.62)	<0.01
**R/R**	91	28	4.31 (2.69–6.89)	<0.01

“R”: D84E, R151C, R160W, D294H, indels, rare variants.

“r”: V60L, V92M, R163Q, R142H, I155T.

### Melanoma Risk Model

To determine the utility of including MC1R genotype and environmental exposure measures (outdoor and indoor UV) in melanoma risk prediction, we first developed a model based on well-established patient phenotypic factors and age (Table 5, Model A), which produced an AUC of 0.72 (95% CI 0.69–0.74). We then separately added outdoor and indoor UV exposure to the model to assess their individual contribution to its predictive ability (Models B and C, respectively). As seen in Table 5, outdoor UV exposure did not significantly improve the model's performance (AUC 0.72, 95% CI = 0.69–0.74; p = 0.66), while the addition of indoor UV exposure resulted in a statistically significant improvement with an AUC of 0.73 (95% CI = 0.70–0.75; p = 0.03). Interestingly, Model D, which includes both outdoor and indoor UV exposure, had the same AUC as Model C (AUC = 0.73, 95% CI = 0.71–0.75; p = 0.02), suggesting that outdoor UV exposure does not contribute to the performance of the model. The addition of MC1R genotype to Model A (Model E) resulted in a statistically significant improvement compared to the baseline model, with an AUC of 0.73 (95% CI = 0.70–0.75; p = 0.02). We interpret this improvement in model performance to be a demonstration of MC1R's functional role outside of hair color (i.e. DNA repair). A full model (Model F) combining phenotypic factors, outdoor and indoor UV exposure, and MC1R genotype performed the best with an AUC of 0.74 (95% CI = 0.71–0.76; p<0.01). These results were consistent in cross-validation analysis, and the models were determined to be well calibrated, as demonstrated by the Hosmer-Lemeshow Goodness-of-fit test with p-values greater than 0.05 (Table 5).

**Table 5:** Description of Melanoma Risk Models.

Model	Variables	All Subjects (N = 1640)		Cross Validation	Hosmer-Lemeshow Goodness-of-Fit test
		AUC (95% CI)	P value*	AUC (95% CI)	P value
**A**	Age	0.72 (0.69–0.74)	Ref	0.70 (0.66–0.75)	0.90
	Gender				
	Hair color				
	Eye color				
	Skin color				
	Freckling				
	Mole phenotype				
**B**	A+total sunburns	0.72 (0.69–0.74)	0.66	0.70 (0.66–0.75)	0.60
**C**	A+hours of indoor tanning	0.73 (0.70–0.75)	0.03	0.71 (0.67–0.76)	0.25
**D**	A+total sunburns+hours of indoor tanning	0.73 (0.71–0.75)	0.02	0.71 (0.67–0.75)	0.08
**E**	A+MC1R Genotype	0.73 (0.70–0.75)	0.02	0.71 (0.66–0.75)	0.69
**F (Full model)**	A+D+E	0.74 (0.71–0.76)	<0.01	0.72 (0.67–0.76)	0.65

* p-values in reference to Model A.

### Testing the Risk Model in Patient Subsets

Since one of the most important phenotypic risk factors for melanoma is number of nevi, we explored the performance of these models in the nevus-resistant and nevus-prone patient subsets, as described in the Methods section (Table 6). The AUC of Model A (baseline model) decreased in both subsets compared to all subjects. The decrease was much more profound in the nevus-prone group compared to the nevus-resistant group (AUC = 0.60 vs. 0.69). In both groups, Model F (final model) performed better than Model A. This incremental improvement was somewhat larger in the nevus-prone group with an increase in AUC from 0.60 to 0.67, compared to the nevus-resistant subgroup with an increase in AUC from 0.69 to 0.72. In analyzing the step-wise improvement of adding UV exposure measures to the baseline Model A, the role of indoor UV exposure (Model C) appears to have a more profound impact on model performance in the nevus-prone subgroup with an increase in AUC from 0.60 to 0.65 compared to the nevus-resistant subgroup with an increase in AUC from 0.69 to 0.71.

**Table 6:** Melanoma Risk Models Stratified by Mole Phenotype.

Model	All patients (N = 1640)		Nevus Resistant* (N = 1321)		Nevus Prone* (N = 319)	
	AUC (95% CI)	P value	AUC (95% CI)	P value	AUC (95% CI)	P value
**A**	0.72 (0.69–0.74)	Ref	0.69 (0.67–0.72)	Ref	0.60 (0.53–0.68)	Ref
**B**	0.72 (0.69–0.74)	0.66	0.70 (0.67–0.72)	0.50	0.61 (0.53–0.68)	0.44
**C**	0.73 (0.70–0.75)	0.03	0.71 (0.68–0.73)	0.09	0.65 (0.57–0.72)	0.03
**D**	0.73 (0.71–0.75)	0.02	0.71 (0.68–0.74)	0.06	0.65 (0.58–0.72)	0.02
**E**	0.73 (0.70–0.75)	0.014	0.70 (0.68–0.73)	0.03	0.62 (0.55–0.70)	0.15
**F (Full model)**	0.74 (0.71–0.76)	<0.01	0.72 (0.69–0.74)	0.01	0.67 (0.60–0.74)	0.01

*The AUC for Nevus resistant and Nevus prone is calculated based on the coefficients obtained from the “All Patient” model.

## Discussion

Our study has several key findings. First, we found that indels and rare variants were associated with a significantly increased melanoma risk. Second, we demonstrated that adding MC1R genotype and indoor UV exposure data to a phenotypic melanoma risk model results in a small, but statistically significant increase in predictive ability, which was upheld when hair color measures were removed from the model (data not shown). The incremental increase in AUC corresponding to adding the predictors is quantitatively small, but it is well known that incremental AUC is a conservative measure of discrimination improvement [25]–[27]. These findings support the potential utility of genetic risk markers to improve the recognition of the more than 50% of melanoma patients that lack common phenotypic risk factors [5], [6], [11], [28]. Lastly, we observed substantial variation in the contribution of indoor UV exposure to the model's performance when subjects were stratified by mole phenotype. This stratification is further supported by interaction analyses suggesting that indoor UV exposure may confer differing increases in melanoma risk between these subgroups (p = 0.06, unpublished data). This suggests that the inherited genetic variants that contribute to the “nevus prone” and “nevus resistant” phenotypes may interact differently with UV exposure to affect melanoma risk.

Whiteman et al. first described the concept that different mole phenotypes may be associated with different melanoma causal pathways as part of “The Divergent Pathway Theory” [29]. This theory provides a conceptual framework connecting the epidemiologic heterogeneity of melanoma with melanoma risk factors, particularly mole phenotype [30]. Published reports demonstrate that nevogenesis has a strong genetic component [9], [10]; therefore, nevus-prone and nevus-resistant individuals likely have different germline genetic variants that influence their mole phenotypes and melanoma risk. Since the number of nevi is extremely important in melanoma risk prediction, stratification by nevus phenotype allowed us to assess whether the additional risk factors (indoor and outdoor UV measures, and MC1R genotype) could improve the model's performance among the lower-risk, nevus-resistant individuals who comprised 72% of the melanoma cases. Although the final model (Model F) performed better in the nevus-resistant compared to the nevus-prone subgroups (AUC = 0.72 vs. 0.67), the degree of improvement from Model A to Model F was somewhat larger in the nevus-prone group. While we recognize the inherent limitations of subgroup analyses, this variation in model performance with the addition of indoor UV exposure provides support for further studies investigating the interaction between environmental exposure and an individual's genetic propensity to develop nevi [31].

Growing evidence supports the utility of targeted cancer screening. In lung cancer, screening a high-risk population led to a significant decrease in mortality [4], [32]. Similarly, a population-based melanoma screening program in Germany was associated with a nearly 50% reduction in melanoma mortality [3]; however, we estimate the number needed to screen to prevent one melanoma death at approximately 127,000. This potentially high cost of population-based melanoma screening could be reduced through a targeted approach similar to that used in lung cancer. The risk assessment model we describe offers one approach to developing a tool to identify individuals for targeted screening, particularly the potential benefit of using genetic information. Adding MC1R genotype resulted in a small, yet significant improvement in the predictive ability of our model. It is also worth noting that this improvement was based on a single gene. In comparison, risk indices for breast and prostate cancers require several genetic markers to produce increases of similar magnitude [22], [24], [28], [33]–[36].

MC1R genotype has been incorporated into two other preliminary risk models [37]–[39]. A Greek hospital-based study incorporated 8 melanoma-related single nucleotide polymorphisms (SNP's), some of which were MC1R variants, into a clinically-based risk model, but investigators did not find a significant improvement in their clinical model's performance with the addition of the SNP's [38]. Most recently, Cust et al. demonstrated that adding MC1R genotypic information increased in the performance of a baseline melanoma risk prediction model, supporting our findings [39]. Of note, their baseline model used age, sex, city of recruitment, and self-reported European ancestry as covariates, which are very different from our baseline model, suggesting that MC1R genotype may be a robust factor to help identify patients at increased risk for melanoma.

Our preliminary melanoma risk model has several strengths compared to existing models [40]–[43]. First, we developed the model using a high-quality, population-based case-control study of over 1600 subjects. Second, we noted a small, yet significant increase in the performance of our model with the addition of UV exposure data, particularly indoor tanning. Third, we characterized each patient for their MC1R genotype, rather than the presence of specific variants. We believe this is a more comprehensive approach as it accounts for both alleles. Finally, we are the first to demonstrate a variation in model performance when stratified by mole phenotype, which suggests that more than one melanoma risk model may be needed to address melanoma etiologic heterogeneity.

In addition to our model's strengths, there are also limitations. The development of a successful melanoma risk model using self-reported host and environmental exposure factors has the benefit of being easily accessible to the general population and potentially cost-effective; conversely, the potential for inaccurate self-assessments of host characteristics (i.e. number of nevi), and recall bias with respect to UV exposure may result in misclassification of melanoma risk. Of note, the risk of recall bias associated with self-reported risk factors was addressed in the parent study. Such bias was analyzed and found not to influence the odds ratios for various phenotypic factors [11]. Secondly, our study (and the parent study) excluded subjects older than age 59 due to the decreasing prevalence of indoor tanning among older patient cohorts. Subsequent studies are needed to test the model in populations that include older individuals. Finally, the small sample size of nevus-prone subjects (n = 319), is a potential limitation of our subgroup analysis, and will need to be validated in a larger sample size. The performance of modified MC1R genotyping may also vary by ancestry and/or geographic locations.

Understandably, our melanoma risk model (Model F) requires replication in additional patient cohorts; however, as a hypothesis-generating model, it is promising that variations in a single gene can significantly improve the predictive performance of a model incorporating host and environmental measures. Very recently, Fang et al. demonstrated that the addition of 11 SNP's identified in melanoma GWAS studies to a basic phenotypic model (i.e. age, gender, hair color, eye color, and skin color) resulted in a 7.8% increase in AUC from 0.64 to 0.69 [44]. This finding supports the role of genetic markers to potentially improve melanoma risk prediction, and suggests that adding these and/or additional genetic markers may further improve the performance of our risk model.

In conclusion, we demonstrated that the inclusion of indoor tanning measures and MC1R genotype improve the predictive ability of a clinically-based melanoma risk model. Variation in the risk model's discriminative ability when applied to phenotypic subgroups suggests that the influence of certain melanoma risk factors may vary by a patient's clinical characteristics, supporting the disease heterogeneity of melanoma as defined by the Divergent Pathway Theory. Finally, the performance improvement by including MC1R genotypes supports existing evidence that genetics can be used to improve melanoma risk prediction.

## Supporting Information

Figure S1 **Diagrams illustrating 4 categories of self-reported nevus density.** Clockwise from top, diagrams 1–4 correspond with having “none”, “few”, “some”, or “many” moles, respectively.
(TIF)

File S1 **Contains Tables S1 and S2.** **Table S1**. Univariate Analysis of Association between Melanoma and Outdoor UV Exposure Variables Collected by the SHS. **Table S2**. Univariate Analysis of Association between Melanoma and the (categorical) Indoor tanning Variables Collected by the SHS.
(DOCX)
