# Supplementary figures and images for: Development of a Melanoma Risk Prediction Model Incorporating MC1R Genotype and Indoor Tanning Exposure: Impact of Mole Phenotype on Model Performance
Source: PLoS One. 2014 Jul 8;9(7):e101507. doi: 10.1371/journal.pone.0101507 (PMC4086828; doi:10.1371/journal.pone.0101507)

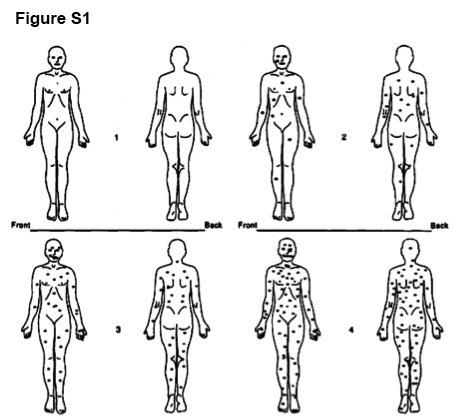

Supplement: Figure S1 — Diagrams illustrating 4 categories of self-reported nevus density. Clockwise from top, diagrams 1–4 correspond with having “none”, “few”, “some”, or “many” moles, respectively. (TIF) [file pone.0101507.s001.tif]
