# Supplementary material for: Development of a Melanoma Risk Prediction Model Incorporating MC1R Genotype and Indoor Tanning Exposure: Impact of Mole Phenotype on Model Performance
Source: PLoS One. 2014 Jul 8;9(7):e101507. doi: 10.1371/journal.pone.0101507 (PMC4086828; doi:10.1371/journal.pone.0101507)
Supplement: File S1 — Contains Tables S1 and S2. Table S1. Univariate Analysis of Association between Melanoma and Outdoor UV Exposure Variables Collected by the SHS. Table S2. Univariate Analysis of Association between Melanoma and the (categorical) Indoor tanning Variables Collected by the SHS. (DOCX) [file pone.0101507.s002.docx]

| **Table S1. Univariate Analysis of Association between Melanoma and Outdoor UV Exposure Variables Collected by the SHS** | | | | |
| --- | --- | --- | --- | --- |
| **Variable name** | **Variable Type** | **Odds Ratio** | **P value** | **95% Confidence interval** |
| **Routine Sun Exposure （per 100-hour）** | | | | |
| Total number of hours | Continuous | 0.994 | 0.04 | 0.989 ~ 0.999 |
| Total number of hours at age 10 | Continuous | 0.968 | 0.02 | 0.941 ~ 0.995 |
| Total number of hours at age 20 | Continuous | 0.991 | 0.33 | 0.972~ 1.009 |
| Total number of hours at age 30 | Continuous | 0.974 | 0.01 | 0.954~ 0.993 |
| Total number of hours at age 40 | Continuous | 0.968 | 0.01 | 0.947~ 0.990 |
| Total number of hours at age 50 | Continuous | 0.985 | 0. 34 | 0.955 ~ 1.016 |
| **Summer Sun Exposure (per 100-hour)** | | | | |
| Total number of hours | Continuous | 0.981 | 0.04 | 0.964 ~ 0.999 |
| Total number of hours at age 10 | Continuous | 0.933 | 0.02 | 0.878 ~ 0.991 |
| Total number of hours at age 20 | Continuous | 0.990 | 0.73 | 0.935 ~ 1.048 |
| Total number of hours at age 30 | Continuous | 0.900 | <0.01 | 0.841 ~ 0.964 |
| Total number of hours at age 40 | Continuous | 0.892 | <0.01 | 0.826 ~ 0.964 |
| Total number of hours at age 50 | Continuous | 0.981 | 0.71 | 0.887 ~ 1.085 |
| **Winter Sun Exposure (per 100-hour)** | | | | |
| Total number of hours | Continuous | 0.992 | 0.05 | 0.985 ~ 1.000 |
| Total number of hours at age 10 | Continuous | 0.965 | 0.07 | 0.929 ~ 1.003 |
| Total number of hours at age 20 | Continuous | 0.985 | 0.25 | 0.960 ~ 1.010 |
| Total number of hours at age 30 | Continuous | 0.970 | 0.03 | 0.944 ~ 0.996 |
| Total number of hours at age 40 | Continuous | 0.962 | 0.01 | 0.933 ~ 0.991 |
| Total number of hours at age 50 | Continuous | 0.978 | 0.28 | 0.938 ~ 1.019 |
| **Recreational and Occupational Sun Exposure (per 100-hour)** | | | | |
| Total hours of 11 outdoor recreational activities | Continuous | 0.994 | 0.04 | 0.989~ 1.000 |
| Total hours of 3 outdoor recreational activities | Continuous | 1.003 | 0.77 | 0.985~ 1.020 |
| Total hours in outdoor jobs in warmer months | Continuous | 0.999 | 0.33 | 0.997 ~ 1.001 |
| Total hours in outdoor jobs in cooler months | Continuous | 0.999 | 0.14 | 0.998~ 1.000 |
| Total hours in the sun across all outdoor jobs | Continuous | 0.999 | 0.18 | 0.999~ 1.000 |
| Total hours of sunny vacation | Continuous | 1.001 | 0.96 | 0.947 ~ 1.059 |
| **Other Measures related to Sun Exposure** | | | | |
| Total number of sunny vacations | Continuous | 1.052 | 0.05 | 1.000 ~ 1.107 |
| Ever been to any sunny vacations | Dichotomous |  |  |  |
| 0=Never (n=254) |  | Reference |  |  |
| 1=Yes (n=1386) |  | 1.240 | 0.12 | 0.949 ~ 1.621 |
| **Outdoor Sunburns** | | | | |
| Painful Sunburn before age 18 | Dichotomous |  |  |  |
| 0=Never (n=204) |  | Reference |  |  |
| 1=Yes (n=1434) |  | 1.636 | <0.01 | 1.216 ~ 2.200 |
| Number of painful sunburns before age 18 | Categorical |  |  |  |
| 0=Never (n=74) |  | Reference |  |  |
| 1=1-2 (n=287) |  | 1.747 | 0.04 | 1.039 ~ 2.935 |
| 2=3-5 (n=358) |  | 1.552 | 0.09 | 0.933 ~ 2.581 |
| 3=6+ (n=717) |  | 2.062 | <0.01 | 1.267 ~ 3.357 |
| Painful Sunburn after the age of 18 | Dichotomous |  |  |  |
| 0=Never (n=204) |  | Reference |  |  |
| 1=Yes (n=1434) |  | 1.756 | <0.01 | 1.326 ~ 2.327 |
| Number of painful sunburns after age 18 | Categorical |  |  |  |
| 0=Never (n=333) |  | Reference |  |  |
| 1=1-2 (n=451) |  | 1.632 | <0.01 | 1.227 ~ 2.172 |
| 2=3-5 (n=396) |  | 1.559 | <0.01 | 1.163 ~ 2.091 |
| 3=6+ (n=445) |  | 1.762 | <0.01 | 1.322 ~ 2.347 |
| Total number of lifetime sunburns | Categorical |  |  |  |
| 0=Never (n=110) |  | Reference |  |  |
| 1=1-2 (n=215) |  | 1.898 | 0.01 | 1.172 ~ 3.076 |
| 2=3-5 (n=317) |  | 2.607 | <0.01 | 1.649 ~ 4.123 |
| 3=6+ (n=995) |  | 2.804 | <0.01 | - 1. ~ 4.269 |

| **Table S2. Univariate Analysis of Association between Melanoma and the (categorical) Indoor tanning Variables Collected by the SHS** | | | | | |
| --- | --- | --- | --- | --- | --- |
| **Variable name** | **Thresholds of the categories** | **Counts of each categories** | **OR** | **95% CI** | **P-value*** |
| Ever use an indoor tanning device | 0=Never Used | 703 | Reference |  |  |
|  | 1=Used | 937 | 1.636 | 1.344 ~ 1.993 | <0.01 |
| Total number of years indoor tanning | 0=Never Used | 703 | Reference |  |  |
|  | 1= ≤1 year | 190 | 1.398 | 1.014 ~ 1.929 | 0.04 |
|  | 2= 2-5 years | 301 | 1.441 | 1.099 ~ 1.891 | 0.01 |
|  | 3= 6-9 years | 155 | 1.782 | 1.250 ~ 2.541 | <0.01 |
|  | 4= 10+ years | 271 | 2.213 | 1.654 ~ 2.962 | <0.01 |
| Total number of indoor tanning sessions | 0=Never Used | 703 | Reference |  |  |
|  | 1= ≤10 sessions | 227 | 1.417 | 1.049 ~ 1.914 | 0.02 |
|  | 2= 11-25 sessions | 173 | 1.476 | 1.056 ~ 2.063 | 0.02 |
|  | 3= 26-100 sessions | 217 | 1.571 | 1.155 ~ 2.137 | <0.01 |
|  | 4= 101+ sessions | 297 | 2.244 | 1.692 ~ 2.976 | <0.01 |
| Total number of hours indoor tanning | 0=Never Used | 703 | Reference |  |  |
|  | 1= 1-9 hours | 462 | 1.437 | 1.135 ~ 1.819 | <0.01 |
|  | 2= 10-19 hours | 93 | 1.234 | 0.800 ~ 1.902 | 0.34 |
|  | 3= 20-49 hours | 152 | 2.040 | 1.419 ~ 2.931 | <0.01 |
|  | 4= 50+ hours | 205 | 2.491 | 1.792 ~ 3.463 | <0.01 |
| Times of indoor tanning sessions per year | 0=Never Used | 703 | Reference |  |  |
|  | 1= 1-5 times/year | 301 | 1.441 | 1.099 ~ 1.891 | 0.01 |
|  | 2= 6-10 times/year | 253 | 1.990 | 1.481 ~ 2.673 | <0.01 |
|  | 3= 11-25 times/year | 205 | 1.420 | 1.039 ~ 1.942 | 0.0 |
|  | 6= 26+ times/year | 155 | 2.291 | 1.590 ~ 3.299 | <0.01 |
| Year of first tan | 0=Never Used | 703 | Reference |  |  |
|  | 1= Year of first tan before 1980 | 125 | 1.735 | 1.178 ~ 2.555 | 0.01 |
|  | 2= Year of first tan between 1980 and 1989 | 410 | 1.770 | 1.382 ~ 2.267 | <0.01 |
|  | 3= Year of first tan between 1990 and 1999 | 313 | 1.649 | 1.260 ~ 2.160 | <0.01 |
|  | 4= Year of first tan after 2000 | 89 | 1.033 | 0.664 ~ 1.607 | 0.88 |
| Number of years since last indoor tan | 0=Never Used | 703 | Reference |  |  |
|  | 1=Current-1 year ago | 257 | 1.280 | 0.961 ~ 1.704 | 0.10 |
|  | 2=2-5 years ago | 250 | 2.369 | 1.750 ~ 3.207 | <0.01 |
|  | 3=6-10 years ago | 144 | 1.666 | 1.158 ~ 2.397 | 0.01 |
|  | 4=11+ years ago | 276 | 1.548 | 1.169 ~ 2.050 | <0.01 |
| Age of first indoor tan | 0=Never Used | 703 | Reference |  |  |
|  | 1= < 18 years old | 255 | 1.441 | 1.099 ~ 1.891 | 0.01 |
|  | 2= 18-24 years old | 207 | 1.990 | 1.481 ~ 2.673 | <0.01 |
|  | 3= 25-34 years old | 217 | 1.420 | 1.039 ~ 1.942 | 0.03 |
|  | 4=35+ years old | 258 | 2.291 | 1.590 ~ 3.299 | <0.01 |
| Ever get a burn from indoor tanning | 0=Never Used | 703 | Reference |  |  |
|  | 5= No | 647 | 1.497 | 1.208 ~ 1.855 | <0.01 |
|  | 10= Yes | 290 | 2.007 | 1.515 ~ 2.659 | <0.01 |
| times burned from indoor tanning | 0=Never Used | 703 | Reference |  |  |
|  | 1= Never burned | 647 | 1.497 | 1.208 ~ 1.855 | <0.01 |
|  | 2= 1 time | 77 | 1.812 | 1.119 ~ 2.932 | 0.02 |
|  | 3= 2 times | 64 | 1.585 | 0.944 ~ 2.660 | 0.08 |
|  | 4 = 3-5 times | 79 | 1.791 | 1.113 ~ 2.880 | 0.02 |
|  | 5 = 6+ times | 70 | 3.341 | 1.916 ~ 5.826 | <0.01 |
| times burned from sun after indoor tanning | 0=Never Used | 703 | Reference |  |  |
|  | 1= Never burned | 689 | 1.563 | 1.265 ~ 1.931 | <0.01 |
|  | 2= 1 time | 78 | 0.991 | 0.620 ~ 1.585 | 0.97 |
|  | 3= 2 times | 58 | 1.638 | 0.952 ~ 2.820 | 0.08 |
|  | 4 = 3-5 times | 54 | 2.517 | 1.391 ~ 4.555 | <0.01 |
|  | 5 = 6+ times | 58 | 4.433 | 2.309 ~ 8.512 | <0.01 |
| Use indoor tanning before 1980 | 0=Never Used | 703 | Reference |  |  |
|  | 1=Prior to 1980 only | 30 | 1.997 | 0.937 ~ 4.259 | 0.07 |
|  | 2=After 1980 only | 776 | 1.622 | 1.320 ~ 1.992 | <0.01 |
|  | 3=Both before and after 1980 | 121 | 1.821 | 1.227 ~ 2.701 | <0.01 |
| Use indoor tanning before 1990 | 0=Never Used | 703 | Reference |  |  |
|  | 1=Prior to 1990 only | 166 | 1.752 | 1.242 ~ 2.473 | <0.01 |
|  | 2=After 1990 only | 367 | 1.463 | 1.135 ~ 1.886 | <0.01 |
|  | 3=Both before and after 1990 | 394 | 1.822 | 1.417 ~ 2.341 | <0.01 |
| ***P-values are computed from the Chi-Square test comparing each group to the “NEVER USE INDOOR TANNING” group of 703 subjects** | | | | | |
